# Glycemic variability in continuous glucose monitoring is inversely associated with baroreflex sensitivity in type 2 diabetes: a preliminary report

**DOI:** 10.1186/s12933-018-0683-2

**Published:** 2018-03-07

**Authors:** Daisuke Matsutani, Masaya Sakamoto, Hiroyuki Iuchi, Souichirou Minato, Hirofumi Suzuki, Yosuke Kayama, Norihiko Takeda, Ryuzo Horiuchi, Kazunori Utsunomiya

**Affiliations:** Division of Diabetes, Metabolism and Endocrinology, Department of Internal Medicine, 3-25-8, Nishi-Shinbashi, Minato-ku, Tokyo, 105-8461 Japan

**Keywords:** Glycemic variability, Baroreflex sensitivity, Cardiovascular autonomic neuropathy, Cardiovascular disease, Continuous glucose monitoring, Type 2 diabetes

## Abstract

**Background:**

It is presently unclear whether glycemic variability (GV) is associated with baroreflex sensitivity (BRS), which is an early indicator of cardiovascular autonomic neuropathy. The present study is the first to examine the relationships between BRS and GV measured using continuous glucose monitoring (CGM).

**Methods:**

This was a multicenter, prospective, open-label clinical trial. A total of 102 patients with type 2 diabetes were consecutively recruited for this study. GV was assessed by measuring the standard deviation (SD), glucose coefficient of variation (CV), and the mean amplitude of glycemic excursions (MAGE) during CGM. The BRS was analyzed from electrocardiogram and blood pressure recordings using the sequence method on the first day of hospitalization.

**Results:**

A total of 94 patients (mean diabetes duration 9.7 ± 9.6 years, mean HbA1c 61.0 ± 16.8 mmol/mol [7.7 ± 1.5%]) were analyzed. In the univariate analysis, CGM-SD (*r *= − 0.375, *p *= 0.000), CGM-CV (*r *= − 0.386, *p *= 0.000), and MAGE (*r *= − 0.395, *p *= 0.000) were inversely related to BRS. In addition to GV, the level of BRS correlated with the coefficient of variation in the R–R intervals (CVR-R) (*r *= 0.520, *p *= 0.000), heart rate (HR) (*r *= − 0.310, *p *= 0.002), cardio-ankle vascular index (CAVI) (*r *= − 0.326, *p *= 0.001), age (*r *= − 0.519, *p *= 0.000), and estimated glomerular filtration rate (eGFR) (*r *= 0.276, *p *= 0.007). Multiple regression analysis showed that CGM-CV and MAGE were significantly related to a decrease in BRS. These findings remained after adjusting the BRS for age, sex, hypertension, dyslipidemia, HR, eGFR, CAVI, and CGM-mean glucose. Additionally, BRS was divided according to quartiles of the duration of diabetes (Q1–4). BRS decreased after a 2-year duration of diabetes independently of age and sex.

**Conclusions:**

GV was inversely related to BRS independently of blood glucose levels in type 2 diabetic patients. Measurement of BRS may have the potential to predict CV events in consideration of GV.

*Trial registration* UMIN Clinical Trials Registry UMIN000025964, 28/02/2017

**Electronic supplementary material:**

The online version of this article (10.1186/s12933-018-0683-2) contains supplementary material, which is available to authorized users.

## Background

Baroreflex sensitivity (BRS), which is a sensitive measure of cardiovascular autonomic neuropathy (CAN) in type 2 diabetic patients [1, 2], is associated with cardiovascular disease events [3–5]. Risk factors involved in the reduced BRS in type 2 diabetes have yet to be fully elucidated. Previously reported risk factors for reduced BRS include hyperglycemia, diabetic duration, older age, obesity, hypoadiponectinemia, arteriosclerosis, and hypertension [6–12]. Among risk factors, although chronic hyperglycemia is an important pathophysiological factor in reduced BRS, improved chronic hyperglycemia alone was not shown to improve CAN in type 2 diabetes [13, 14]. In addition to chronic hyperglycemia, increased glycemic variability (GV) may be an independent risk factor for developing CAN [15–19]. However, the relationship between GV and BRS has not been clarified. GV was previously evaluated by self-monitoring of blood glucose (SMBG) 7 times a day, mainly in insulin-treated patients. The recent clinical application of continuous glucose monitoring (CGM) and flash glucose monitoring has allowed detailed assessment of GV [20].

The present study is the first to examine the relationships between BRS and GV measured using CGM.

## Methods

### Study participants

This was a multicenter, prospective, open-label clinical trial. A total of 102 patients with type 2 diabetes (69 males and 33 females) were consecutively recruited from hospitalized patients at Jikei University School of Medicine Hospital, Tokyo, Japan and Tsuruoka kyoritsu Hospital, Yamagata, Japan. Inclusion criteria were age ≥ 20 years and the presence of type 2 diabetes diagnosed according to 2017 American Diabetes Association guidelines. Exclusion criteria were taking non-dihydropyridine calcium channel blockers, digitalis, or antipsychotics. Exclusion criteria also included arrhythmia, severe renal dysfunction (serum Cr ≥ 2.5 mg/dL), severe liver dysfunction (3X upper limit of normal), severe infection, severe trauma, pre- and post-operation, diabetic ketosis, diabetic coma, insulin-dependent diabetes mellitus, malignancy, myocardial infarction, heart failure, cerebral infarction within a half year, pregnancy, or heavy alcohol consumption.

**Fig. 1:**
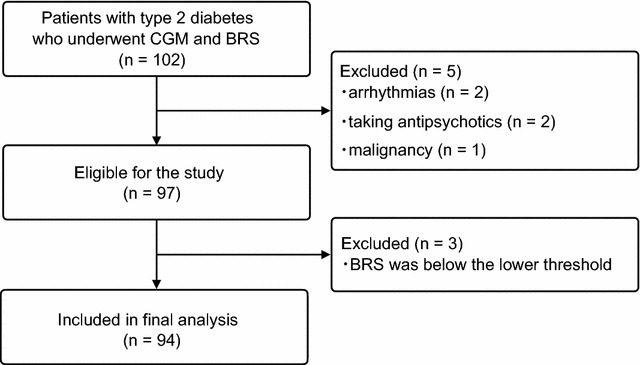
Study population. Ninety-four participants were enrolled in this study. *CGM* continuous glucose monitoring, *BRS* baroreflex sensitivity

Of the 102 recruited patients, 97 were included in the analysis after excluding 2 with arrhythmias, 2 patients who were taking antipsychotics, and 1 with a malignancy. Further excluded were 3 participants whose BRS was below the lower threshold. Finally, a total of 94 patients with type 2 diabetes (66 males and 28 females) were analyzed (Fig. 1).

All participants underwent blood tests, including those for fasting plasma glucose (FPG), HbA1c, estimated glomerular filtration rate (eGFR), high density lipoprotein-cholesterol (HDL-C), low density lipoprotein-cholesterol (LDL-C), and triglycerides. Clinical data (duration of diabetes, body mass index [BMI], systolic blood pressure [SBP], diastolic blood pressure [DBP], insulin therapy, use of an oral anti-diabetic drugs, lipid-lowering agents and anti-hypertensive agents, smoking experience) were obtained from medical records and a questionnaire. Dyslipidemia and hypertension were diagnosed based on Japanese guidelines. Dyslipidemia was defined as HDL-C < 40 mg/dL, LDL-C ≥ 140 mg/dL, and/or triglycerides ≥ 150 mg/dL) and/or taking at least one lipid-lowering agent. Hypertension was defined as SBP ≥ 140 mmHg and/or DBP ≥ 90 mmHg and/or taking at least one anti-hypertensive agent. The study protocol was approved by the local ethics committee, and the study was conducted according to the principles of the Helsinki Declaration II. All patients were informed of the purpose of the study after which consent was obtained.

### Assessment of glycemic variability

**Fig. 2:**
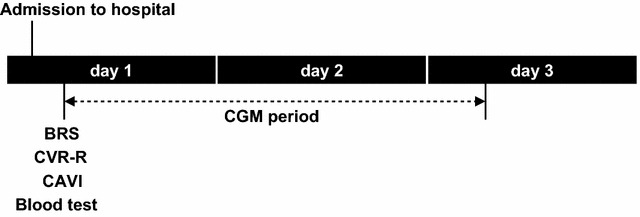
Study protocol. On admission, a blood test was performed under fasting conditions. BRS, CVR-R, and CAVI were evaluated on the first day of hospitalization. Subcutaneous interstitial glucose levels were monitored over a period of 2 consecutive days using continuous glucose monitoring (CGM). *BRS* baroreflex sensitivity, *CVR-R* coefficient of variation in the R–R intervals, *CAVI* cardio-ankle vascular index

All study patients were monitored continuously with a continuous glucose monitor (ipro-2, Medtronic, Minneapolis, MN, USA) for 48 h beginning the first hospital day (Fig. 2). The monitor’s sensor was inserted into abdominal subcutaneous adipose tissue and calibrated according to the standard Medtronic ipro-2 operating guidelines. During the monitoring, patients measured their blood glucose at least 4 times per day with a SMBG device (Medisafe FIT, Terumo, Tokyo, Japan). After 48 h of monitoring, the glucose profile and GV parameters were analyzed with computer software. GV was assessed by measuring the standard deviation (SD), glucose coefficient of variation (CV), and the mean amplitude of glycemic excursions (MAGE) during the CGM. CV was calculated by dividing the SD by the mean of the corresponding glucose readings, and the MAGE was calculated by measuring the arithmetic mean of the differences between consecutive peaks and nadirs, provided that the differences were greater than one SD of the mean glucose value. Patients continued anti-hyperglycemic therapy as usual and avoided glucose infusions during CGM.

### Assessment of baroreflex sensitivity

BRS was evaluated on the first day of hospitalization (Fig. 2). Beat-to-beat BP was measured for 15 min after 15 min of supine rest by the spontaneous sequence method as the slope of the relationship between spontaneous changes in SBP and the pulse interval [21, 22]. Beat-to-beat BP was measured using the second and third fingers of the right hand by the vascular unloading technique (Task Force Monitor, CNSystems, Graz, Austria). The system was calibrated using a conventional noninvasive BP cuff wrapped around the left upper arm. Calibration was performed automatically every 2 min. A standard 3-lead electrocardiogram (ECG) was used to record the heart rate (HR). For calculation of BRS, the relative changes in BP (mmHg) and the R–R interval (ms), which is expressed as the distance between corresponding QRS complexes, were considered according to the sequence method with cut-off points of 1 mmHg and 3 ms, respectively.

### Assessment of the CV in the R–R intervals (CVR-R)

The CVR-R was measured based on previously reported methods by the Cardio Star FCP-7431 (Fukuda Denshi, Tokyo, Japan) [23]. CVR-R was recorded by taking 100 samples of the HR on the first day of hospitalization (Fig. 2). Based on the mean R–R intervals (mRR) and the R–R standard deviation (RR-SD), the CVR-R was calculated as RR-SD/mRR × 100 (%) (reference range > 2%).

### Assessment of the cardio-ankle vascular index (CAVI)

The CAVI was measured by the VaSera VS-3000N (Fukuda Denshi, Tokyo, Japan) on the first day of hospitalization (Fig. 2). With participants in the supine position, BP was measured at the brachial artery, and heart beats were monitored by an ECG. The length from the aortic valve to the ankle and the time for the pulse wave to propagate from the heart to the ankle were measured. The principle of the CAVI formula and its calculation were described previously [24]. For statistical evaluation of the CAVI, mean values for the left and right sides were used.

### Statistical analyses

Data analyses were performed using the Statistical Package for the Social Sciences 22.0 software (IBM, Armonk, NY, USA). Patient characteristics and results are presented as mean ± SD. Pearson’s correlation analysis was used for single correlations. Multiple-linear regression was used to assess individual and cumulative effects of GV (CGM-SD, CGM-CV, and MAGE), age, sex, hypertension, dyslipidemia, HR, eGFR, CAVI, and CGM-mean glucose on BRS. Independent variables were selected based on previous studies pertaining to factors associated with low levels of BRS [10, 25–28]. Diabetes durations were divided into quartiles (Q1 < 2, Q2 ≥ 2 to  < 7, Q3 ≥ 7 to  < 14, Q4 ≥ 14 years) (Tables 5, 6, Fig. 5). The analysis of variance (ANOVA) was used to compare BRS among participants according to quartiles of diabetes duration. The Jonckheere trend test was used to test for linear trends in BRS in relation to diabetes duration quartiles. In ANOVA, the Games–Howell post hoc test was also used to compare the BRS results among different diabetes duration groups. In the multivariate analysis, analysis of covariance (ANCOVA) was used to compare the coefficients of BRS among different diabetes duration groups with adjustment for age (years) and sex (male vs. female). In ANCOVA, the Bonferroni post hoc tests were also used to compare the BRS results among different diabetes duration groups. The Student’s t test or the non-parametric Mann–Whitney U-test was used to compare the means of continuous variables. A *p* < 0.05 was considered significant.

## Results

### Baseline characteristics of study participants

A total of 94 patients were analyzed. Clinical and anthropometric characteristics of the study participants are shown in Table 1. Baseline drug administration is shown in Table 2. The prevalence of study participants ever diagnosed with hypertension or dyslipidemia was 72 and 88%, respectively. The mean age of participants was 62.1 ± 11.9 years, mean duration of diabetes was 9.7 ± 9.6 years, and mean HbA1c was 61.0 ± 16.8 mmol/mol (7.7 ± 1.5%). At baseline, 27% of patients were on sulfonylureas, 13% on insulin, 36% on renin–angiotensin–aldosterone system (RAAS) inhibitors (angiotensin-converting enzyme inhibitors and/or angiotensin receptor blockers), 34% on calcium-channel blockers, and 5% on beta-blockers, and 30% on a statins.

**Table 1:** Baseline patient characteristics

Baseline data	
No. of patients	94
Sex, male/female	66/28
Age (years)	62.1 ± 11.9
Body mass index (kg/m^2^)	26.2 ± 5.0
Duration of diabetes (years)	9.7 ± 9.6
Fasting plasma glucose (mg/dL)	141.8 ± 36.4
HbA1c (mmol/mol)	61.0 ± 16.8
HbA1c (%)	7.7 ± 1.5
Smoking, n (%)	30 (32)
Hypertension, n (%)	68 (72)
Dyslipidemia, n (%)	83 (88)
Blood pressure (mmHg)	
Systolic	121.9 ± 17.2
Diastolic	76.5 ± 9.9
Heart rate (beats/min)	69.2 ± 11.0
Lipid profile (mg/dL)	
Triglycerides	150.2 ± 87.0
LDL-cholesterol	112.9 ± 29.0
HDL-cholesterol	50.4 ± 15.3
eGFR (ml/min/1.73 m^2^)	76.4 ± 16.6
CGM parameters (mg/dL)	
Mean glucose	156.7 ± 31.6
SD	35.1 ± 13.4
CV	22.4 ± 7.5
MAGE	88.2 ± 32.2
BRS (ms/mmHg)	9.0 ± 4.5
CVR-R (%)	2.9 ± 1.4
CAVI	8.5 ± 1.2

Values are mean ± SD or no. (%)

*LDL* low density lipoprotein, *HDL* high density lipoprotein, *eGFR* estimated glomerular filtration rate, *CGM* continuous glucose monitoring, *SD* standard deviation, *CV* coefficient of variance, *MAGE* mean amplitude of glycemic excursions, *BRS* baroreflex sensitivity, *CVR-R* coefficient of variation in the R–R intervals, *CAVI* cardio-ankle vascular index

**Table 2:** Baseline drug administration

Therapy	
Oral anti-diabetic drugs, n (%)	69 (73)
Metformin, n (%)	35 (37)
Sulfonylureas, n (%)	25 (27)
Thiazolidinediones, n (%)	10 (11)
Glinides, n (%)	3 (3)
DPP-4 inhibitors, n (%)	46 (49)
α-glucosidase inhibitors, n (%)	5 (5)
SGLT2 inhibitors, n (%)	11 (12)
GLP-1 receptor agonists, n (%)	3 (3)
Insulin, n (%)	12 (13)
Anti-hypertensive agents, n (%)	45 (48)
RAAS inhibitors, n (%)	34 (36)
Calcium-channel blockers, n (%)	32 (34)
Thiazides, n (%)	5 (5)
Beta-blockers, n (%)	5 (5)
Lipid-lowering agents, n (%)	32 (34)
Statins, n (%)	28 (30)

Values are no. (%)

*DPP* dipeptidyl peptidase-4, *SGLT* sodium glucose cotransporter, *GLP* glucagon-like peptide, *RAAS* renin–angiotensin–aldosterone system

### Univariate correlates of BRS

**Fig. 3:**
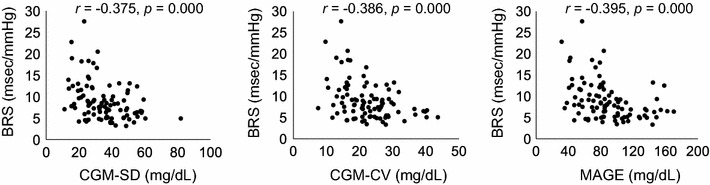
Relationship between BRS and glycemic variability. *BRS* baroreflex sensitivity, *CGM* continuous glucose monitoring, *SD* standard deviation, *CV* coefficient of variance, *MAGE* mean amplitude of glycemic excursions

**Fig. 4:**
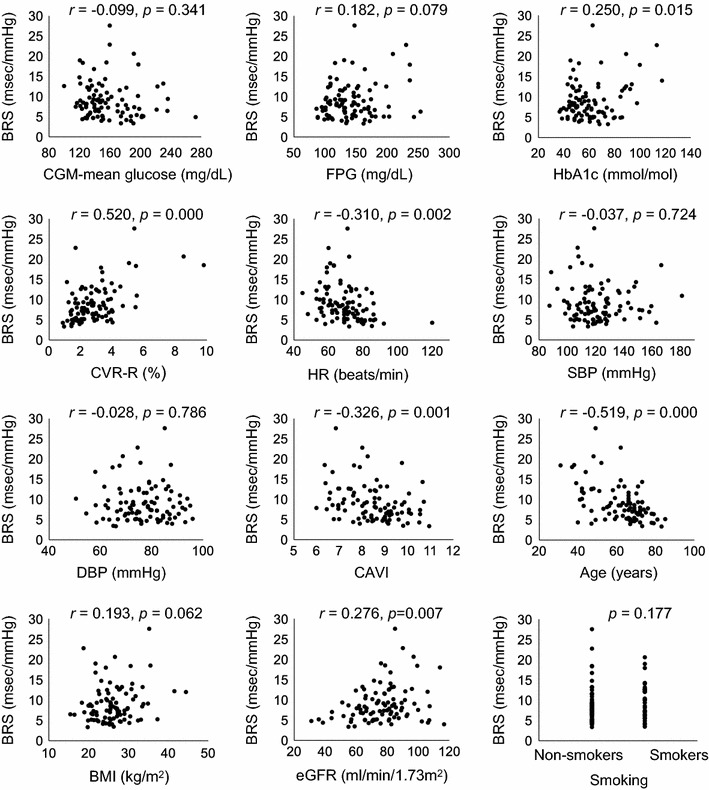
Univariate correlates of BRS. *BRS* baroreflex sensitivity, *CGM* continuous glucose monitoring, *FPG* fasting plasma glucose, *CVR-R* coefficient of variation in the R–R intervals, *HR* heart rate, *SBP* systolic blood pressure, *DBP* diastolic blood pressure, *CAVI* cardio-ankle vascular index, *BMI* body mass index, *eGFR* estimated glomerular filtration rate

Correlation analysis showed that parameters of GV, such as the of CGM-SD (*r *= − 0.375, *p *= 0.000), CGM-CV (*r *= − 0.386, *p *= 0.000), and MAGE (*r *= − 0.395, *p *= 0.000), were significantly related to low levels of BRS (Fig. 3, Table 3). In addition to GV, the level of BRS correlated with CVR-R (*r *= 0.520, *p *= 0.000), HR (*r *= − 0.310, *p *= 0.002), CAVI (*r *= − 0.326, *p *= 0.001), age (*r *= − 0.519, *p *= 0.000), and eGFR (*r *= 0.276, *p *= 0.007). However, CGM-mean glucose (*r *= − 0.099, *p *= 0.341), FPG (*r *= 0.182, *p *= 0.079), HbA1c (*r *= 0.250, *p *= 0.015), SBP (*r *= − 0.037, *p *= 0.724), DBP (*r *= − 0.028, *p *= 0.786), and BMI (*r *= 0.193, *p *= 0.062) did not (Table 3, Fig. 4).

**Table 3:** Univariate correlates of BRS

Variables	*r*	*p*
CGM-SD (mg/dL)	− 0.375	0.000
CGM-CV (mg/dL)	− 0.386	0.000
MAGE (mg/dL)	− 0.395	0.000
CGM-mean glucose (mg/dL)	− 0.099	0.341
FPG (mg/dL)	0.182	0.079
HbA1c (mmol/mol)	0.250	0.015
CVR-R (%)	0.520	0.000
HR (beats/min)	− 0.310	0.002
SBP (mmHg)	− 0.037	0.724
DBP (mmHg)	− 0.028	0.786
CAVI	− 0.326	0.001
Age (years)	− 0.519	0.000
BMI (kg/m^2^)	0.193	0.062
eGFR (mL/min/1.73 m^2^)	0.276	0.007
Triglycerides (mg/dL)	0.171	0.099
LDL-cholesterol (mg/dL)	0.165	0.111
HDL-cholesterol (mg/dL)	− 0.094	0.365

*BRS* baroreflex sensitivity, *CGM* continuous glucose monitoring, *SD* standard deviation, *CV* coefficient of variance, *MAGE* mean amplitude of glycemic excursions, *FPG* fasting plasma glucose, *CVR-R* coefficient of variation in the R–R intervals, *HR* heart rate, *SBP* systolic blood pressure, *DBP* diastolic blood pressure, *CAVI* cardio-ankle vascular index, *BMI* body mass index, *eGFR* estimated glomerular filtration rate, *LDL* low density lipoprotein, *HDL* high density lipoprotein

### Multivariate analysis of BRS

Multiple regression analysis showed that CGM-CV and MAGE were inversely related to BRS. These findings remained after adjusting BRS for age, sex, hypertension, dyslipidemia, HR, eGFR, CAVI, and CGM-mean glucose. In addition to GV, age, HR, and CAVI were found to be predictive factors for BRS (Table 4).

**Table 4:** Multiple regression analysis of BRS

Dependent variables	Model 1		Model 2	
	*β*	*p*	*β*	*p*
(a)				
CGM-SD (mg/dL)	− 0.218	0.051	− 0.336	0.004
Age (years)	− 0.435	0.000	–	–
Sex (male/female)	− 0.029	0.731	− 0.061	0.506
Hypertension	− 0.073	0.418	− 0.087	0.369
Dyslipidemia	− 0.183	0.038	− 0.166	0.080
Heart rate (beats/min)	− 0.259	0.004	− 0.244	0.012
eGFR (mL/min/1.73 m^2^)	0.035	0.714	0.138	0.160
CAVI	–	–	− 0.216	0.028
CGM-mean glucose (mg/dL)	0.119	0.240	0.199	0.066
(b)				
CGM-CV (mg/dL)	− 0.206	0.032	− 0.304	0.002
Age (years)	− 0.427	0.000	–	–
Sex (male/female)	− 0.031	0.711	− 0.063	0.492
Hypertension	− 0.071	0.428	− 0.084	0.385
Dyslipidemia	− 0.184	0.036	− 0.166	0.077
Heart rate (beats/min)	− 0.257	0.004	− 0.243	0.012
eGFR (mL/min/1.73 m^2^)	0.042	0.657	0.149	0.125
CAVI	–	–	− 0.207	0.035
CGM-mean glucose (mg/dL)	0.012	0.889	0.034	0.721
(c)				
MAGE (mg/dL)	− 0.227	0.036	− 0.346	0.002
Age (years)	− 0.414	0.000	–	–
Sex (male/female)	− 0.020	0.809	− 0.044	0.630
Hypertension	− 0.088	0.328	− 0.110	0.256
Dyslipidemia	− 0.165	0.055	− 0.139	0.131
Heart rate (beats/min)	− 0.274	0.002	− 0.269	0.005
eGFR (mL/min/1.73 m^2^)	0.035	0.712	0.135	0.166
CAVI	–	–	− 0.179	0.074
CGM-mean glucose (mg/dL)	0.119	0.231	0.192	0.069

The dependent variable was BRS, and the independent variables were Model 1 and Model 2. Model 1: adjustment for GV, age, sex, hypertension, dyslipidemia, heart rate, eGFR, and CGM-mean glucose; Model 2: adjustment for GV, sex, hypertension, dyslipidemia, heart rate, eGFR, CAVI, and CGM-mean glucose; GV was (a) CGM-SD, (b) CGM-CV, and (c) MAGE. Model 1 (a) R-squared 0.414, adjusted R-squared 0.359; (b) R-squared 0.420, adjusted R-squared 0.365; (c) R-squared 0.418, adjusted R-squared 0.364; Model 2 (a) R-squared 0.321, adjusted R-squared 0.257, (b) R-squared 0.328, adjusted R-squared 0.264, (c) R-squared 0.329, adjusted R-squared 0.266

*BRS* baroreflex sensitivity, *CGM* continuous glucose monitoring, *SD* standard deviation, *CV* coefficient of variance, *MAGE* mean amplitude of glycemic excursions, *eGFR* estimated glomerular filtration rate, *CAVI* cardio-ankle vascular index

### Comparisons of BRS according to duration of diabetes

**Fig. 5:**
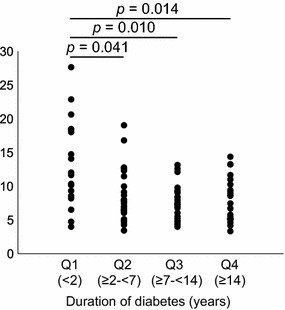
Relationship between BRS and duration of diabetes based on ANOVA. Baroreflex sensitivity divided according to quartiles of the duration of diabetes. The Games–Howell post hoc test compared with Q1

Figure 5 and Table 5 show the comparisons of BRS among participants with various durations of diabetes according to quartiles based on ANOVA. There was a significant difference in BRS among these four groups. The results were then analyzed by the Games–Howell post hoc test. The Q2 (*p *= 0.041), Q3 (*p *= 0.010), and Q4 (*p *= 0.014) groups had reduced BRS in comparison with the Q1 group. This observation was confirmed by the Jonckheere trend test: BRS (*p *= 0.005) was significantly correlated with the quartiles of diabetes duration. For the multivariate analysis, Table 6 shows the comparisons of BRS among participants with various quartiles of diabetes duration based on ANCOVA. The results were then analyzed by the Bonferroni post hoc test.

**Table 5:** Comparisons of BRS according to duration of diabetes based on ANOVA

Diabetes duration (quartiles)	Q1 (< 2)	Q2 (≥ 2 to < 7)	Q3 (≥ 7 to < 14)	Q4 (≥ 14)	ANOVA	Test for trend
	(n = 18)	(n = 28)	(n = 24)	(n = 24)	*p* value	*p* value
BRS (ms/mmHg)	13.3 ± 6.5	8.5 ± 3.7*	7.6 ± 2.6*	7.7 ± 3.2*	0.010	0.005
*p* value		0.041	0.010	0.014		
HbA1c (mmol/mol)	69 ± 25.6	57.2 ± 13.6	58.8 ± 12.7	61.7 ± 14.6		
HbA1c (%)	8.5 ± 2.3	7.4 ± 1.2	7.5 ± 1.2	7.8 ± 1.3		
CGM-mean glucose (mg/dL)	152.1 ± 23.9	153.1 ± 36.1	162.1 ± 32.6	159.2 ± 31.8		
CGM-SD (mg/dL)	30.7 ± 12.2	33 ± 12.1	33.5 ± 13.3	42.6 ± 13.7		
CGM-CV (mg/dL)	20.3 ± 8.1	21.8 ± 7.8	20.2 ± 5.6	26.7 ± 6.9		
MAGE (mg/dL)	76.5 ± 30.6	85.7 ± 30.2	85.4 ± 34.9	102.6 ± 29.7		

Values are mean ± SD. BRS was divided according to quartiles of diabetes duration. The Games–Howell post hoc test compared with Q1: * *p* < 0.05

**Table 6:** Comparison of BRS according to diabetes duration based on ANCOVA

Diabetes duration (quartiles)	Q1 (< 2)	Q2 (≥ 2 to < 7)	Q3 (≥ 7 to < 14)	Q4 (≥ 14)	ANCOVA
	(n = 18)	(n = 28)	(n = 24)	(n = 24)	*p* value
BRS (ms/mmHg)	12.1 ± 0.9	8.3 ± 0.8*	7.6 ± 0.8*	8.1 ± 1.0*	0.003
*p* value		0.012	0.003	0.03	
HbA1c (mmol/mol)	69 ± 25.6	57.2 ± 13.6	58.8 ± 12.7	61.7 ± 14.6	
HbA1c (%)	8.5 ± 2.3	7.4 ± 1.2	7.5 ± 1.2	7.8 ± 1.3	
CGM-mean glucose (mg/dL)	152.1 ± 23.9	153.1 ± 36.1	162.1 ± 32.6	159.2 ± 31.8	
CGM-SD (mg/dL)	30.7 ± 12.2	33 ± 12.1	33.5 ± 13.3	42.6 ± 13.7	
CGM-CV (mg/dL)	20.3 ± 8.1	21.8 ± 7.8	20.2 ± 5.6	26.7 ± 6.9	
MAGE (mg/dL)	76.5 ± 30.6	85.7 ± 30.2	85.4 ± 34.9	102.6 ± 29.7	

Values for BRS are adjusted mean ± SE, all other values are mean ± SD. BRS was divided according to quartiles of diabetes duration. Adjustment for age (years) and sex (male vs. female). The Bonferroni post hoc test compared with Q1: * *p* < 0.05

After adjustment for age and sex, the Q2 (*p *= 0.012), Q3 (*p *= 0.003), and Q4 (*p *= 0.030) groups had significantly reduced BRS compared with the Q1 group.

### Comparisons of BRS according to various subgroups

No difference existed in BRS between hypertensive and normotensive participants (8.5 ± 4.4 vs. 10.4 ± 4.6 ms/mmHg, *p* = 0.056) (Additional file 1: Tables S1 and S2), those with dyslipidemia and normal lipid values (8.8 ± 4.4 vs. 10.0 ± 5.8 ms/mmHg, *p* = 0.424), and the presence or absence of a smoking history (9.9 ± 4.5 vs. 8.5 ± 4.5 ms/mmHg, *p* = 0.177). Use of sulfonylurea was associated with low levels of BRS compared with its non-use (sulfonylurea use vs. non-use: 7.1 ± 3.2 vs. 9.7 ± 4.8 ms/mmHg, *p* = 0.015) (Table 7). The GV in patients taking sulfonylurea was larger than in those who did not (sulfonylurea use vs. non-use: CGM-SD 41.9 ± 14.0 vs. 32.7 ± 12.5 mg/dL, *p *= 0.003; CGM-CV 25.5 ± 8.6 vs. 21.3 ± 6.8 mg/dL, *p *= 0.015; MAGE 102.8 ± 30.2 vs. 82.9 ± 31.5 mg/dL, *p *= 0.007) (Additional file 1: Table S3). There was no significant relationship between the mean BRS and the use of insulin, RAAS inhibitors (angiotensin-converting-enzyme inhibitors and/or angiotensin II receptor blockers), calcium-channel blockers, beta-blockers, or statins (Table 7).

**Table 7:** Comparison of BRS in various subgroups

Condition or therapy	No. (%)	BRS (ms/mmHg)	*p* value for interaction
Hypertension			
Yes	68 (72)	8.5 ± 4.4	0.056
No	26 (28)	10.4 ± 4.6	
Hyperlipidemia			
Yes	83 (88)	8.8 ± 4.4	0.424
No	11 (12)	10.0 ± 5.8	
Smoking			
Yes	30 (32)	9.9 ± 4.5	0.177
No	64 (68)	8.5 ± 4.5	
Sulfonylurea use			
Yes	25 (27)	7.1 ± 3.2	0.015
No	69 (73)	9.7 ± 4.8	
Insulin use			
Yes	12 (13)	9.7 ± 5.6	0.556
No	82 (87)	8.9 ± 4.4	
RAAS inhibitor use			
Yes	34 (36)	8.6 ± 4.8	0.284
No	60 (64)	9.2 ± 4.4	
Calcium-channel blocker use			
Yes	32 (34)	8.5 ± 4.8	0.258
No	62 (66)	9.2 ± 4.4	
Beta-blocker use			
Yes	5 (5)	8.4 ± 3.7	0.788
No	89 (95)	9.0 ± 4.6	
Statin use			
Yes	28 (30)	9.0 ± 5.0	0.138
No	66 (70)	9.0 ± 4.4	

Values are mean ± SD or no. (%)

*BRS* baroreflex sensitivity, *RAAS* renin–angiotensin–aldosterone system

## Discussion

This is the first study of type 2 diabetic patients to examine the relationship between BRS and GV as measured by CGM.

Results showed that CGM-SD, CGM-CV, and MAGE were inversely related to BRS. However, no inverse correlations were found between BRS and the levels of CGM-mean glucose, FPG, and HbA1c. The multiple regression analysis also showed that CGM-CV and MAGE were predictors of BRS independent of age, sex, hypertension, dyslipidemia, HR, eGFR, CAVI, and CGM-mean glucose.

As in previous reports, our analysis also showed that age, HR, and CAVI were predictors of BRS [10, 29]. Additionally, BRS decreased after a 2-year duration of diabetes. Further analysis of the association of oral anti-diabetic drugs with BRS indicated that sulfonylurea was associated with reduced BRS.

Although it was previously reported that BRS was decreased in type 2 diabetic patients [6, 30], the detailed mechanism has not been elucidated. In this study, GV, which is an independent risk factor for developing CV events [31–36], was inversely related to BRS independent of CGM-mean glucose. This result suggests that GV is involved in the reduced BRS in type 2 diabetes rather than chronic hyperglycemia. Our study further supports existing data showing that individuals with impaired glucose tolerance (IGT) had reduced BRS compared to individuals with normal glucose tolerance (NGT), while it was previously shown that those with impaired fasting glycemia (IFG) did not have reduced BRS [19, 37]. On the other hand, oral anti-diabetic drugs have a relatively large effect on GV. In this study, patients taking sulfonylurea had larger GV than those who did not (sulfonylurea use vs. non-use: CGM-SD 41.9 ± 14.0 vs. 32.7 ± 12.5 mg/dL, *p *= 0.003; CGM-CV 25.5 ± 8.6 vs. 21.3 ± 6.8 mg/dL, *p *= 0.015; MAGE 102.8 ± 30.2 vs. 82.9 ± 31.5 mg/dL, *p *= 0.007) (Additional file 1: Table S3). Furthermore, sulfonylureas were associated with reduced BRS (*p *= 0.015). It was reported that increased blood levels of insulin are also involved in reduced BRS [38]. Although the present study did not examine insulin levels, increased insulin levels may explain this phenomenon.

The following findings suggested that GV causes the reduced BRS by increasing oxidative stress and inducing endothelial dysfunction independently of chronic hyperglycemia: (1) GV increases oxidative stress [39–41] and oxidative stress causes neuropathy [42, 43]; and (2) GV induces endothelial dysfunction [40, 41] and endothelial dysfunction causes neuropathy [44]. In addition to GV, our study showed that CAVI was independently correlated with BRS. Although it was reported that arteriosclerosis evaluated by intima-media thickness or pulse wave velocity was involved in the reduced BRS [11, 27], this is the first report showing the relationship between CAVI and BRS.

The point during the course of diabetes at which BRS reduction begins to occur remains controversial [45]. We found that BRS began to decrease in patients after a relatively short duration of diabetes. In patients with type 2 diabetes of a short duration, increased GV caused by postprandial hyperglycemia is the predominant pathophysiological feature [46, 47]. Therefore, in our patients with diabetes of a short duration, an increased GV may have led to an early reduction in BRS.

This study has three notable limitations. First, the cross-sectional design without a control group does not allow for causal relationships to be identified. Hence, the observed associations may only serve as hypothesis generating. Further prospective studies are necessary to clarify causal relationships. Second, although BRS has been reported to decrease with the progression of CAN, the present study excluded patients whose BRS was less than the lower threshold. Moreover, although this study used the sequence method, it did not use other traditional methods. Third, the duration of hypertension or dyslipidemia was not taken into consideration.

## Conclusions

GV was inversely related to BRS independently of blood glucose levels in type 2 diabetic patients. Although further prospective studies are necessary to clarify the causal relationship, these results may suggest that GV is an important risk factor affecting BRS.

## Additional file

**Additional file 1: Table S1.** Comparison of clinical characteristics among subjects with hypertensive and normotensive. **Table S2.** Univariate correlates of BRS in subjects with hypertensive and normotensive. **Table S3.** Comparison of BRS in subgroups

## Abbreviations

BRS: baroreflex sensitivity
CAN: cardiovascular autonomic neuropathy
CAVI: cardio-ankle vascular index
CGM: continuous glucose monitoring
CVD: cardiovascular disease
CVR-R: coefficient of variation in the R–R intervals
GV: glycemic variability
