# Supplementary material for: Glycemic variability in continuous glucose monitoring is inversely associated with baroreflex sensitivity in type 2 diabetes: a preliminary report
Source: Cardiovasc Diabetol. 2018 Mar 7;17:36. doi: 10.1186/s12933-018-0683-2 (PMC5840775; doi:10.1186/s12933-018-0683-2)
Supplement: Supplementary file 1 — Additional file 1: Table S1. Comparison of clinical characteristics among subjects with hypertensive and normotensive. Table S2. Univariate correlates of BRS in subjects with hypertensive and normotensive. Table S3. Comparison of BRS in subgroups [file 12933_2018_683_MOESM1_ESM.docx]

| **Table S1.** Comparison of clinical characteristics among subjects with hypertensive and normotensive | | | | | | | |
| --- | --- | --- | --- | --- | --- | --- | --- |
| Baseline data | Normotensive | | | Hypertensive | | | *p* |
| No. of patients | 26 | | | 68 | | |  |
| Sex, male/female | 15/11 | | | 51/17 | | | 0.101 |
| Age (years) | 59.5 | ± | 13.0 | 63.1 | ± | 11.3 | 0.188 |
| Body mass index (kg/m^2^) | 23.8 | ± | 3.8 | 27.1 | ± | 5.2 | 0.004 |
| Duration of diabetes (years) | 9.1 | ± | 9.8 | 10.0 | ± | 9.6 | 0.691 |
| Fasting plasma glucose (mg/dL) | 138.1 | ± | 38.6 | 143.3 | ± | 35.5 | 0.542 |
| HbA1c (mmol/mol) | 71.2 | ± | 20.6 | 62.0 | ± | 15.7 | 0.774 |
| HbA1c (%) | 7.7 | ± | 1.8 | 7.7 | ± | 1.4 | 0.774 |
| Smoking, n (%) | 6 (6) | | | 24 (26) | | | 0.239 |
| Hypertension, n (%) | 0 (0) | | | 68 (72) | | | 0.000 |
| Hyperlipidemia, n (%) | 22 (23.4) | | | 60 (64) | | | 0.732 |
| Blood pressure (mmHg) |  |  |  |  |  |  |  |
| Systolic | 109.2 | ± | 10.7 | 127.3 | ± | 16.7 | 0.000 |
| Diastolic | 70.4 | ± | 9.0 | 79.2 | ± | 8.8 | 0.000 |
| Heart rate (beats/min) | 66.3 | ± | 9.1 | 48.0 | ± | 12.5 | 0.117 |
| Lipid profile (mg/dL) |  |  |  |  |  |  |  |
| Triglycerides | 157.0 | ± | 105.4 | 147.3 | ± | 79.7 | 0.647 |
| LDL-cholesterol | 116.5 | ± | 26.8 | 111.3 | ± | 30.0 | 0.470 |
| HDL-cholesterol | 54.8 | ± | 17.1 | 48.4 | ± | 14.3 | 0.079 |
| eGFR (ml/min/1.73m^2^) | 83.4 | ± | 14.6 | 73.5 | ± | 16.7 | 0.011 |
| CGM parameters (mg/dL) |  |  |  |  |  |  |  |
| Mean glucose | 146.8 | ± | 25.3 | 160.7 | ± | 33.2 | 0.060 |
| SD | 32.3 | ± | 13.6 | 35.9 | ± | 12.9 | 0.203 |
| CV | 21.8 | ± | 7.2 | 22.4 | ± | 7.3 | 0.645 |
| MAGE | 85.1 | ± | 31.8 | 89.1 | ± | 32.3 | 0.572 |
| BRS (msec/mmHg) | 10.4 | ± | 4.6 | 8.5 | ± | 4.4 | 0.056 |
| CVR-R (%) | 2.6 | ± | 1.0 | 3.0 | ± | 1.6 | 0.311 |
| CAVI | 8.3 | ± | 1.0 | 8.6 | ± | 1.3 | 0.224 |
| Values are mean ± SD or no. (%). The categorical variables of the two groups were compared using the chi-square test. The Student’s t-test or the non-parametric Mann-Whitney U-test was used to compare the means of continuous variables. A *p* value < 0.05 was considered significant. LDL, low density lipoprotein; HDL, high density lipoprotein; eGFR, estimated glomerular filtration rate; CGM, continuous glucose monitoring; SD, standard deviation; CV, coefficient of variance; MAGE, mean amplitude of glycemic excursions; BRS, baroreflex sensitivity; CVR-R, coefficient of variation in the R-R intervals; CAVI, cardio-ankle vascular index | | | | | | | |

| **Table S2.** Univariate correlates of BRS in subjects with hypertensive and normotensive | | | | |
| --- | --- | --- | --- | --- |
|  | Normotensive | | Hypertensive | |
| Variables | *r* | *p* | *r* | *p* |
| CGM-SD (mg/dL) | -0.382 | 0.054 | -0.347 | 0.004 |
| CGM-CV (mg/dL) | -0.471 | 0.015 | -0.351 | 0.004 |
| MAGE (mg/dL) | -0.466 | 0.016 | -0.359 | 0.003 |
| CGM-mean glucose (mg/dL) | 0.066 | 0.749 | -0.100 | 0.421 |
| FPG (mg/dL) | 0.490 | 0.011 | 0.073 | 0.559 |
| HbA1c (mmol/mol) | 0.365 | 0.067 | 0.205 | 0.096 |
| CVR-R (%) | 0.267 | 0.187 | 0.645 | 0.000 |
| HR (beats/min) | -0.302 | 0.134 | -0.287 | 0.019 |
| SBP (mmHg) | -0.133 | 0.516 | 0.126 | 0.311 |
| DBP (mmHg) | -0.154 | 0.453 | 0.140 | 0.258 |
| CAVI | -0.251 | 0.217 | -0.331 | 0.006 |
| Age (years) | -0.434 | 0.027 | -0.539 | 0.000 |
| BMI (kg/m^2^) | 0.040 | 0.845 | 0.338 | 0.005 |
| eGFR (ml/min/1.73m^2^) | 0.170 | 0.406 | 0.261 | 0.033 |
| BRS, baroreflex sensitivity; CGM, continuous glucose monitoring; SD, standard deviation; CV, coefficient of variance; MAGE, mean amplitude of glycemic excursions; FPG, fasting plasma glucose; CVR-R, coefficient of variation in the R-R intervals; HR, heart rate; SBP, systolic blood pressure; DBP, diastolic blood pressure; CAVI, cardio-ankle vascular index; BMI, body mass index; eGFR, estimated glomerular filtration rate | | | | |

| **Table S3.** Comparison of BRS in subgroups | | | | | | | | | | | | | | | | | | | | | |  |
| --- | --- | --- | --- | --- | --- | --- | --- | --- | --- | --- | --- | --- | --- | --- | --- | --- | --- | --- | --- | --- | --- | --- |
| Subgroup | No (%) | CGM-SD (mg/dL) | | | *p* value | CGM-CV (mg/dL) | | | *p* value | | MAGE (mg/dL) | | | | *p* value | | BRS (msec/mmHg) | | | | *p* value | |
| Hypertension |  |  |  |  |  |  |  |  | |  | |  |  |  | |  |  |  | |  |  | |
| Yes | 68 (72) | 36.2 | ± | 13.2 | 0.203 | 22.6 | ± | 7.6 | | 0.645 | | 89.4 | ± | 32.3 | | 0.572 | 8.4 | ± | | 4.4 | 0.056 | |
| No | 26 (28) | 32.3 | ± | 13.9 |  | 21.8 | ± | 7.3 | |  |  | 85.1 | ± | 32.4 | |  | 10.4 | ± | | 4.7 |  |  |
| Dyslipidemia |  |  |  |  |  |  |  |  | |  | |  |  |  | |  |  |  | |  |  | |
| Yes | 83 (88) | 34.1 | ± | 12.4 | 0.049 | 21.7 | ± | 6.9 | | 0.018 | | 86.6 | ± | 30.3 | | 0.180 | 8.8 | ± | | 4.4 | 0.424 | |
| No | 11 (12) | 42.6 | ± | 18.6 |  | 27.4 | ± | 10.1 | |  |  | 100.5 | ± | 44.4 | |  | 10.0 | ± | | 5.8 |  |  |
| Smoking |  |  |  |  |  |  |  |  | |  | |  |  |  | |  |  |  | |  |  | |
| Yes | 30 (32) | 34.2 | ± | 12.7 | 0.665 | 20.6 | ± | 5.7 | | 0.121 | | 88.2 | ± | 29.8 | | 0.999 | 9.9 | ± | | 4.5 | 0.177 | |
| No | 64 (68) | 35.5 | ± | 13.8 |  | 23.2 | ± | 8.1 | |  |  | 88.2 | ± | 33.6 | |  | 8.5 | ± | | 4.5 |  |  |
| Insulin |  |  |  |  |  |  |  |  | |  | |  |  |  | |  |  |  | |  |  | |
| Yes | 12 (13) | 38.7 | ± | 19.3 | 0.321 | 22.7 | ± | 9.3 | | 0.880 | | 83.6 | ± | 38.0 | | 0.601 | 9.7 | ± | | 5.6 | 0.556 | |
| No | 82 (87) | 34.6 | ± | 12.4 |  | 22.4 | ± | 7.3 | |  |  | 88.8 | ± | 31.5 | |  | 8.9 | ± | | 4.4 |  |  |
| Sulfonylurea |  |  |  |  |  |  |  |  | |  | |  |  |  | |  |  |  | |  |  | |
| Yes | 25 (27) | 41.9 | ± | 14.0 | 0.003 | 25.5 | ± | 8.6 | | 0.015 | | 102.8 | ± | 30.2 | | 0.007 | 7.1 | ± | | 3.2 | 0.015 | |
| No | 69 (73) | 32.7 | ± | 12.5 |  | 21.3 | ± | 6.8 | |  |  | 82.9 | ± | 31.5 | |  | 9.7 | ± | 4.8 | |  |  |
| Statin |  |  |  |  |  |  |  |  | |  | |  |  |  | |  |  |  |  | |  | |
| Yes | 28 (30) | 34.7 | ± | 11.2 | 0.824 | 21.7 | ± | 5.5 | | 0.495 | | 89.9 | ± | 28.4 | | 0.743 | 9.0 | ± | 5.0 | | 0.138 | |
| No | 66 (70) | 35.3 | ± | 14.4 |  | 22.7 | ± | 8.2 | |  |  | 87.5 | ± | 33.9 | |  | 9.0 | ± | 4.4 | |  |  |
| RAAS inhibitor |  |  |  |  |  |  |  |  | |  | |  |  |  | |  |  |  |  | |  | |
| Yes | 34 (36) | 35.1 | ± | 12.8 | 0.985 | 21.6 | ± | 6.9 | | 0.459 | | 86.5 | ± | 33.4 | | 0.703 | 8.6 | ± | 4.8 | | 0.284 | |
| No | 60 (64) | 35.2 | ± | 13.9 |  | 22.8 | ± | 7.8 | |  |  | 89.1 | ± | 31.8 | |  | 9.2 | ± | 4.4 | |  |  |
| CCB |  |  |  |  |  |  |  |  | |  | |  |  |  | |  |  |  |  | |  | |
| Yes | 32 (34) | 32.7 | ± | 12.0 | 0.217 | 20.4 | ± | 5.8 | | 0.057 | | 80.6 | ± | 29.3 | | 0.100 | 8.5 | ± | 4.8 | | 0.258 | |
| No | 62 (66) | 36.4 | ± | 14.0 |  | 23.4 | ± | 8.1 | |  |  | 92.1 | ± | 33.2 | |  | 9.2 | ± | 4.4 | |  |  |
| Beta-blockers |  |  |  |  |  |  |  |  | |  | |  |  |  | |  |  |  |  | |  | |
| Yes | 5 (5) | 29.0 | ± | 7.3 | 0.301 | 18.9 | ± | 5.0 | | 0.291 | | 62.0 | ± | 10.9 | | 0.062 | 8.4 | ± | 3.7 | | 0.788 | |
| No | 89 (95) | 35.5 | ± | 13.6 |  | 22.6 | ± | 7.6 | |  |  | 89.6 | ± | 32.4 | |  | 9.0 | ± | 4.6 | |  |  |
| Values are mean ± SD or no. (%). BRS, baroreflex sensitivity; RAAS, renin-angiotensin-aldosterone system; CCB, calcium-channel blockers | | | | | | | | | | | | | | | | | | | | | |  |
